# Protocol for RNA fluorescence *in situ* hybridization in mouse meningeal whole mounts

**DOI:** 10.1016/j.xpro.2022.101256

**Published:** 2022-03-22

**Authors:** Olof Rickard Nilsson, Laszlo Kari, Rebecca Rosenke, Olivia Steele-Mortimer

**Affiliations:** 1 Laboratory of Bacteriology, Rocky Mountain Laboratories, National Institute of Allergy and Infectious Diseases, National Institutes of Health, Hamilton, MT 59840-2932, USA; 2 Rocky Mountain Veterinary Branch, Rocky Mountain Laboratories, National Institute of Allergy and Infectious Diseases, National Institutes of Health, Hamilton, MT 59840-2932, USA

**Keywords:** Immunology, Microscopy, Antibody, In Situ Hybridization, Neuroscience

## Abstract

The multilayered meninges surrounding the brain and spinal cord harbor distinct immune cell populations with prominent roles in health and diseases. Here we present an optimized protocol for RNA fluorescence *in situ* hybridization (RNA FISH) in meningeal whole mounts, allowing the visualization of gene expression. We also describe the combination of this protocol with immunohistochemistry for simultaneous visualization of mRNA and proteins. This protocol can be used for assessing spatial gene expression within the meninges.

## Highlights

• A protocol for the isolation of mouse meninges and preparation of dried whole mounts
• Optimization of whole mount *in situ* hybridization for assessing spatial gene expression
• RNA FISH protocol can be combined with immunohistochemistry
• Allows simultaneous visualization of gene expression and protein localization

## Graphical abstract

**Figure:**
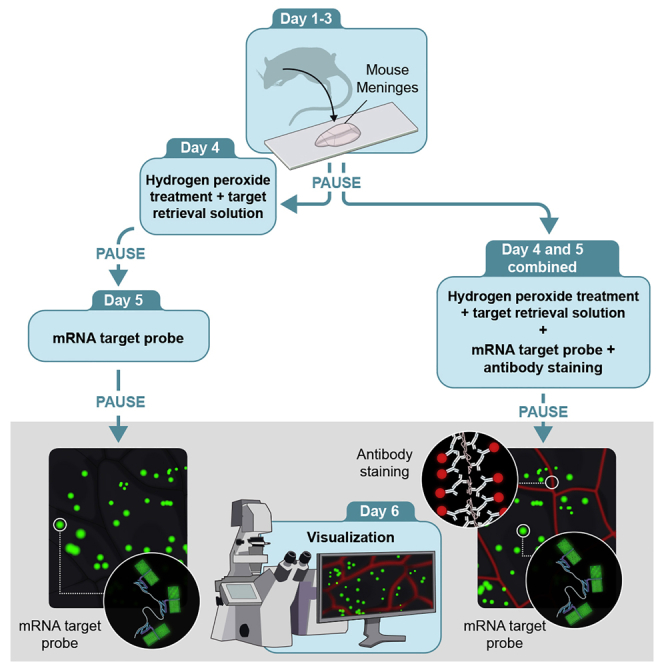

## Before you begin

In this protocol, we describe the steps for preparing whole mounts of mouse meninges and the specific steps for *in situ* hybridization assay (ISH) for detection of target RNA within the meninges. We use the commercially available RNAscope technology (Wang et al., 2012), an ISH based on a probe design by Advanced Cell Diagnostics (https://acdbio.com), that optimizes signal amplification and background noise suppression. Here we use probes to *Pecam1* (*Cd31*), *Aif1*-C3 (*Iba1*) and *Cx3cr1* to target macrophages and endothelial cells in the meninges. We also demonstrate that, with additional optimization, immunostaining with antibodies to specific proteins can be multiplexed in the tissue.

All animal experiments in this study adhered to the Guide for Care and Use of Laboratory Animals, 8^th^ edition (National Research Council). Animal protocols were reviewed and approved by the Rocky Mountain Laboratories Animal Care and Use Committee (Animal Study Protocol number 2019-043).

### Order target RNA probes and opal reagents

**Timing: Up to 3 weeks**

1. A wide variety of probes can be ordered from the ACD catalog and are usually shipped within 2 weeks. Alternatively, probes can be Made-to-Order, but will take longer.
2. Select the correct combination of Opal reagents for detection of probes. One fluorophore must be assigned to each probe channel (C1, C2 or C3). In this protocol, we have successfully used Opal 520 (green), Opal 570 (red) and Opal 690 (far red) reagents. Make sure your microscope is set up correctly for viewing the selected fluorophores. Additional guidance for fluorophore selection can be found at https://acdbio.com/rnascope-fluorescent-multiplex-assay. A list of available Opal Fluorophore reagent packs can be found at https://www.akoyabio.com/phenoimager/assays/.

### Practice extraction of meninges

**Timing: 2 weeks**

3. It is recommended to practice the collection of the skull cap, extraction and mounting of meninges before beginning any ISH experiment. This is to ensure high quality samples with properly preserved structure.
  a. Following O/N fixation, but prior to meningeal extraction, calvaria can be split down the midline using a razor blade, yielding two pieces for practicing extraction and mounting of meninges.

## Key resources table

**Table:** 

REAGENT or RESOURCE	SOURCE	IDENTIFIER
**Antibodies**		

Rabbit anti-Mouse CD31 (1:1000)	Abcam	Cat# ab124432 RRID: AB_2802125
Rabbit anti-Rat IBA1 (1:1000)	Wako	Cat# 019-19741 RRID: AB_839504
Goat anti-Rabbit IgG, HRP-conjugated (1:2000)	Cell Signaling Technology	Cat# 7074 RRID: AB_2099233

**Chemicals, peptides, and recombinant proteins**		

Opal 520 Reagent Pack	Akoya Biosciences	Cat# FP1487001KT
Opal 570 Reagent Pack	Akoya Biosciences	Cat# FP1488001KT
Opal 690 Reagent Pack	Akoya Biosciences	Cat# FP1497001KT
RNAscope 3-Plex Positive Control Probe-Mm	ACD	Cat# 320881
RNAscope 3-Plex Negative Control Probe-Mm	ACD	Cat# 320871
RNAscope probe-Mm-Pecam1	ACD	Cat# 316721
RNAscope probe-Mm-Aif1-C3	ACD	Cat# 319141-C3
RNAscope probe-Mm-Cx3cr1	ACD	Cat# 314221
10% Neutral Buffered Formalin	Cancer Diagnostics	Cat# FX1000
Sterile Pharmaceutical Grade Saline	Vet One	Cat# NDC 13985-807-50
Heparin	Sagent Pharmaceuticals	Cat# NDC 25021-400-30
Phosphate Buffered Saline	Rocky Mountain Laboratories	
Trizma Base	Sigma-Aldrich	Cat# T6066-1KG
Bovine Serum Albumin	Calbiochem	Cat# 12659
ProLong Glass Antifade Mountant	Invitrogen	Cat# P36980
Tween-20	BIO-RAD	Cat# 1706531

**Critical commercial assays**		

RNAscope Multiplex Fluorescent Reagent Kit v2	ACD	Cat# 323100

**Experimental models: Organisms/strains**		

RML mice, (2–12 months), male or female	Rocky Mountain Laboratories	N/A
B6.129P2(Cg)-*Cx3cr1*^*tm1Litt*^/J (*Cx3cr1*^*GFP*^) mice, (6–12 weeks), male or female (CX3CR1 KO mice)	JAX	Cat# 005582
B6.129(Cg)-*Ccr2*^*tm2.1lfc*^/J (*Ccr2*^*RFP*^) mice, (6–12 weeks), male or female (CCR2 KO mice)	JAX	Cat# 017586
B6.129(Cg)F1-*Cx3cr1*^*tm1Litt*^*Ccr2*^*tm2.1lfc*^ mice, (6–12 weeks), male or female (CX3CR1 heterozygous mice)	Rocky Mountain Laboratories	N/A

**Software and algorithms**		

Fiji (ImageJ)	Schneider et al. (2012)	https://imagej.net/software/fiji/
SVI Huygens Essential	Scientific Volume Imaging	https://svi.nl/HomePage

**Other**		

ImmEdge Hydrophobic Barrier Pen	ACD	Cat# 310018
HybEz II Hybridization System	ACD	Cat# 321710
TOMO Adhesion Microscope Slides	Matsunami	Cat# TOM-11/90
Vegetable Steamer, 5.5 quart	Hamilton Beach	Model 37530A
Tissue Tek staining dish	Sakura Finetek	Cat# 4457
Tissue Tek slide holder	Sakura Finetek	Cat# 4465
Fine Scissors	Fine Science Tools	Cat# 14958-11
Spring Scissors	Fine Science Tools	Cat# 15024-10
Graefe Forceps	Fine Science Tools	Cat# 11050-10
Dumont #7 Forceps (fine)	Fine Science Tools	Cat# 11271-30
Nikon Eclipse *Ti2* fluorescence microscope	Nikon	https://www.microscope.healthcare.nikon.com/products/inverted-microscopes/eclipse-ti2-series
Nikon SMZ1500 stereomicroscope (dissection microscope)	Nikon	https://www.microscopyu.com/museum/model-smz1500-stereomicroscope

## Materials and equipment

***Alternatives:*** This protocol uses a Hamilton Beach 5.5 quart vegetable steamer, model 37530A, for performing the target retrieval. However, other steamers can be used.

***Alternatives:*** For RNA FISH involving 1–3 targets qualitative analysis can be done using most research grade epi-fluorescence wide field microscopes with a CCD or CMOS camera or confocal microscopes. We used a Nikon *Ti2* widefield fluorescence microscope equipped with an Orca-Flash 4.0 sCMOS camera (Hamamatsu Photonics). Specific computer software, e.g., ImageJ (Fiji) (Schneider et al., 2012), is required for post-acquisition analysis.

**Table:** Buffers

Buffer name	Components
TBST (TBS wash buffer)	TBS + 0.005% Tween-20 (v/v).
TBSB	TBS + 0.1% Bovine serum albumin (w/v).
1× wash buffer	50× ACD wash buffer diluted 1:50 in distilled water.
1× target retrieval solution	10× ACD target retrieval solution diluted 1:10 in distilled water.

**Table:** PBS, pH 7.4

Reagent	Final concentration	Amount
NaCl	137 mM	8 g
KCl	2.7 mM	0.2 g
Na_2_HPO_4_	10 mM	1.42 g
KH_2_PO_4_	1.8 mM	0.24 g
ddH_2_O	n/a	1 L
**Total**	**n/a**	**1 L**

**Table:** TBS, pH 7.6

Reagent	Final concentration	Amount
NaCl	150 mM	8.766 g
Tris base	50 mM	6.057 g
HCl, 6 M	n/a	To pH 7.6
ddH_2_O	n/a	1 L
**Total**	**n/a**	**1 L**

50× wash buffer and 10× target retrieval solution are supplied in the RNAscope Multiplex Fluorescent Reagent Kit v2, ACD, #323100.

RNAse-free reagents are not required for this protocol.

All buffers are stored at room temperature (RT; in our lab 20°C–21°C) for up to one year.

## Step-by-step method details

### Preparation of dried meningeal whole mounts

**Timing: 3 days**

Meninges are fixed in situ, extracted, and dried onto microscope slides to prepare them for RNA FISH.

**Figure 1:**
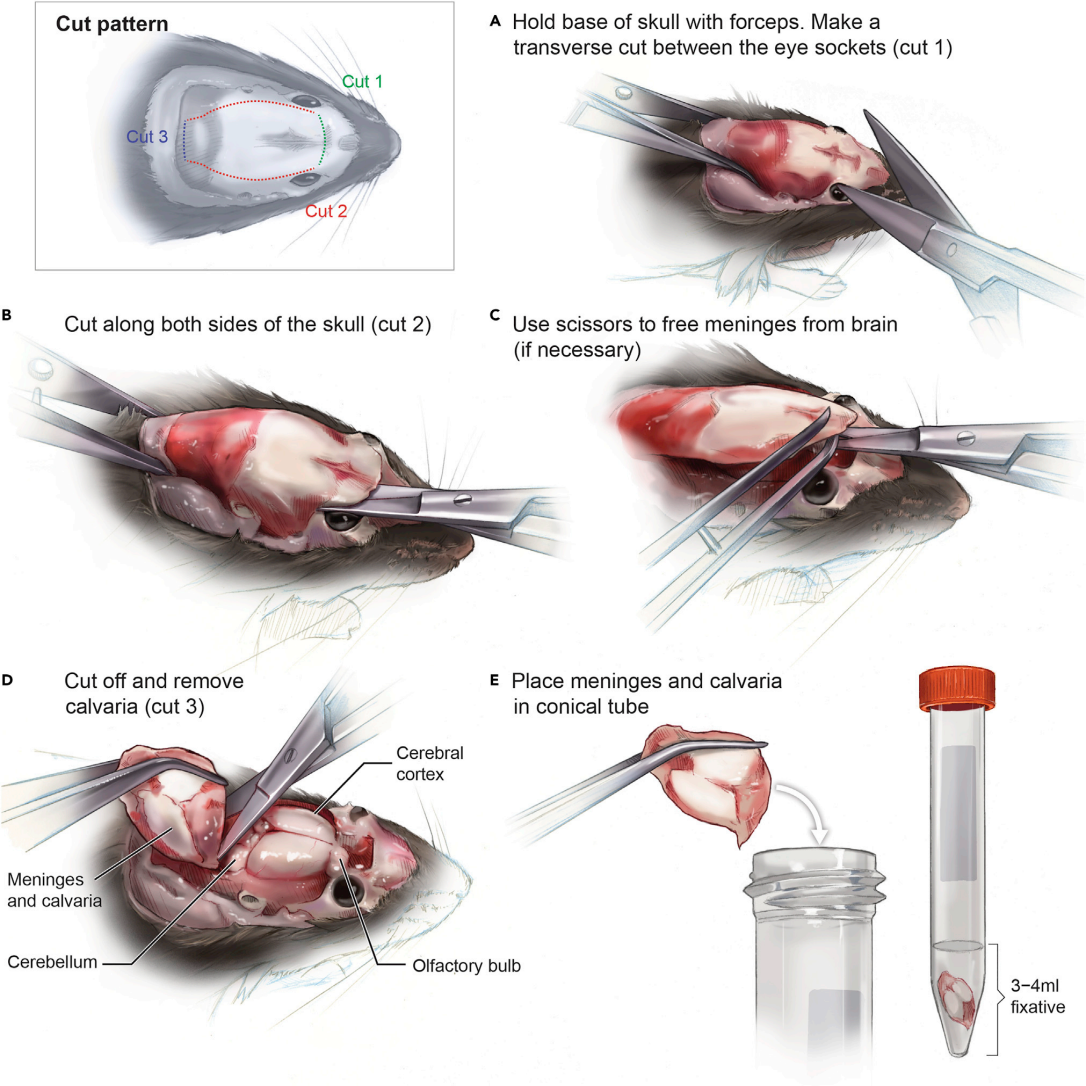
Illustration describing the harvest of calvaria containing meninges from euthanized mice Top left panel indicates the cut sequence. Panels A–E illustrate each step of the harvest of the calvaria. See also Methods video S1.

***Note:*** When removing skull caps, pay close attention to make sure the meninges are extracted along with the skull cap. Sometimes the meninges will come loose, remaining associated with the brain. If this happens, use spring scissors to carefully separate the meninges from the brain while removing the skull cap (Figure 1C).

***Note:*** Complete perfusion is vital for an optimal RNA FISH result, as red blood cells are highly autofluorescent. Perfusion is effective when muscle twitches and blanching of the liver are observed. For more information, see troubleshooting problem 1.

***Note:*** While this protocol uses 10% formalin for fixation, we have used 4% paraformaldehyde with similar results. If performing immunohistochemistry, fixation with 4% paraformaldehyde is preferred as formalin fixation can reduce immunoreactivity of target proteins with antibodies. If the fixation time, typically 18–20 h, is changed adjustments may have to be made to protease digestion duration to facilitate proper penetration of target probes into the tissue. Lowering the fixation temperature, e.g., by fixation at 4°C, may also increase target binding of some antibodies, by better preservation of antibody epitopes.

1. Harvest calvaria (skull cap) containing meninges and fix O/N (Louveau et al., 2015, 2018) (Figure 1):
  a. Following euthanasia, immediately transcardially perfuse mice with 10 mL sterile pharmaceutical grade saline containing heparin (100 U/mL) followed by 6 mL 10% neutral buffered formalin.
  b. Using fine scissors, cut away fur and skin covering the skull. While holding mouse down firmly with a pair of forceps grasping around the neck, make a transverse cut between the eye sockets using fine scissors (Figure 1A).
  c. Using spring scissors, carefully cut posteriorly along the sides of the skull, starting at the transverse cut made in 1b (Figure 1B).
  d. Finally, use a pair of forceps to lift off the calvaria with meninges attached (Figures 1C and 1D). Fix calvaria with associated meninges in 10% neutral buffered formalin O/N at RT in a 15 mL conical tube.

2. The next day, extract the fixed meninges from the calvaria (Methods video S1):
  a. Prepare a 24 well plate with two wells containing 1 mL of PBS for each meninges.
  b. Briefly rinse a calvaria in PBS and place it under a stereomicroscope (optional but recommended). Make sure meninges do not dry out by adding approximately 100 μL PBS.
  c. Extract the meningeal layers from the skull cap using fine forceps (Louveau et al., 2015, 2018) by carefully loosening the meninges around the perimeter. Then, starting at the olfactory bulb area, gently start peeling off meninges. If areas of the meninges stay attached, carefully loosen them with the forceps before continue pulling. Peel meninges towards the midline of the calvaria. Once meninges are gathered in the middle, pull meninges loose, starting at the cerebellum and ending at the olfactory bulbs.
  d. Place extracted meninges in the first well with PBS. Wash meninges for 5 min on a shaker (70 rpm).
  e. Using forceps, gently transfer meninges to the second well containing PBS and wash for another 5 min on a shaker (70 rpm). Meninges can be left in PBS for some time (e.g., if mounting several meninges sequentially), but should not be stored in the PBS for prolonged periods of time (maximum 1 h at RT).

***Note:*** To facilitate testing of new probes or antibodies, split the calvaria down the midline using a razor blade before extracting the meninges. This generates two samples from each calvaria.

3. Mount extracted meninges onto microscope slides (Methods video S2):
  a. Place approximately 100 μL PBS in the middle of a microscope slide. Add the washed meninges to the droplet.
  b. Using fine forceps, gently spread meninges out. Carefully remove some of the liquid using a pipette and spread meninges out further. When meninges are sufficiently spread out, remove as much as possible of the remaining liquid using a paper tissue.
  c. Dry meninges on a flat surface O/N at RT.

**Figure 2:**
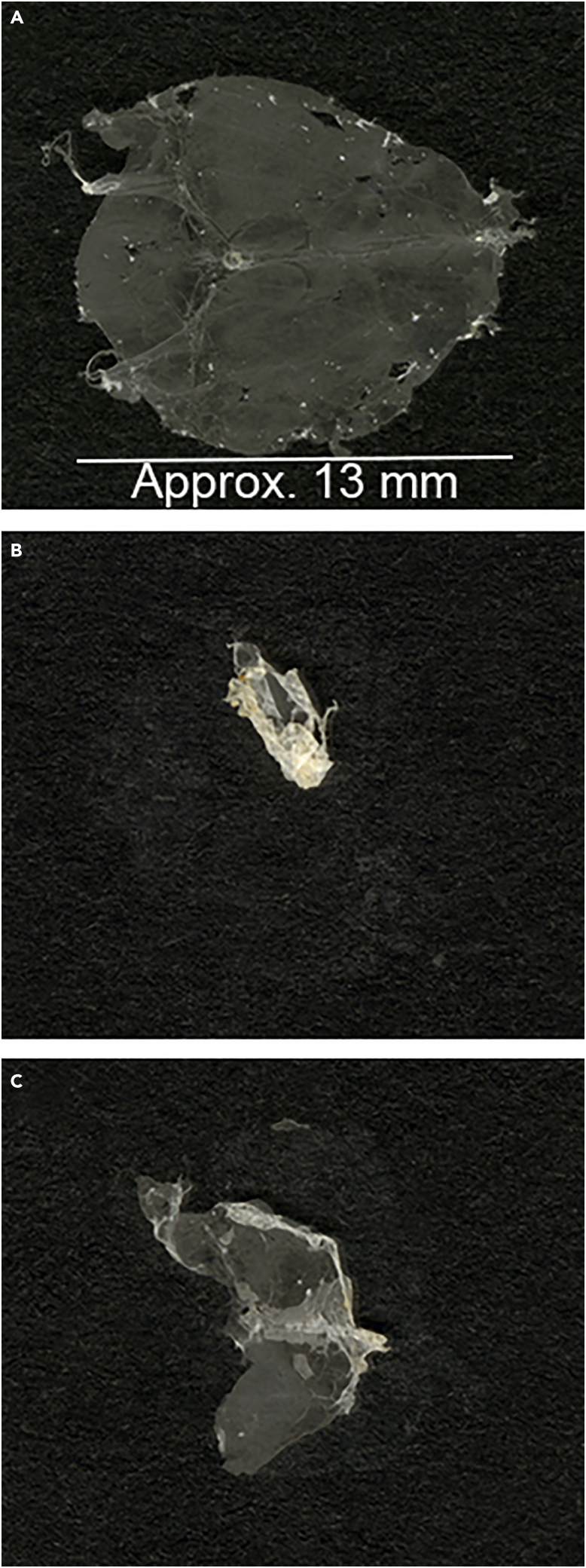
Images of meningeal whole mounts dried onto microscope slides Examples of meningeal whole mount properly dried onto a microscope slide (A) and of improperly dried whole mounts after target retrieval (B and C). Due to insufficient drying, meninges detach during the target retrieval process. Scale bar indicates approximate size of a whole mount, 13 mm. See also Methods video S2.

**CRITICAL:** Proper drying of meninges is crucial for subsequent steps (Figure 2). Improperly dried meninges will detach from slides during target retrieval. Drying time depends on the type of microscope slide. The drying times listed above were optimized using Matsunami TOMO adhesion microscope slides. For more information, see troubleshooting problem 2.

4. The next day, wash dried meninges by dipping them about 10 times in distilled water. Dry meninges for 24 h at 42°C or for at least 3 days at RT.

**Pause point:** Once meninges have been dried, they can be stored at RT in a dry place. We have successfully used samples that were stored for up to 1 year.

Methods video S1. Extraction of meninges from calvaria, related to step 2

Methods video S2. Mounting of extracted meninges onto microscope slides, related to step 3

### Run the RNA FISH assay on dried meningeal whole mounts

**Timing: 7–9 h depending on the number of probes**

Dried meninges are subjected to the RNA FISH procedure to visualize targeted mRNA.

This protocol follows the manufacturer’s protocol, with a few modifications, and can be found using the following link (document #323100-USM).

https://acdbio.com/sites/default/files/USM-323100%20Multiplex%20Fluorescent%20v2%20User%20Manual_10282019_0.pdf

5. Set up equipment for target retrieval: Add 1 L distilled water to the water chamber of a Hamilton Beach vegetable steamer and put one container with 200 mL distilled water and one container with 200 mL 1× target retrieval solution in the steaming compartment. Start the preset steaming program, setting the duration for at least 1 h. Use a thermometer to monitor the temperature of the target retrieval solution.

***Note:*** It may be difficult to reach the target temperature of 100°C for the target retrieval solution. In our laboratory, located at an altitude of approx. 3,500 feet, the maximum temperature was 94°C.

6. Lay slide with dried meninges flat on the bench. When the target retrieval solution in the steamer reaches 90°C, add 4–5 drops of hydrogen peroxide to meninges and incubate for 10 min at RT. Briefly wash meninges by placing slides in a Tissue Tek slide holder and dipping them in a Tissue Tek staining dish filled with distilled water. Repeat wash. Keep slides in the Tissue Tek slide holder. By now the target retrieval solution should have reached the target temperature.
7. Carefully open the steamer and submerge slides in the water for 15 s. Transfer slides to the target retrieval solution and incubate for 10–15 min. Carefully remove slides from the steamer and rinse them in a Tissue Tek staining dish filled with distilled water for 15 s and then incubate them in a Tissue Tek staining dish filled with 100% ethanol for 3 min at RT.

***Optional:*** If a steamer is not available, target retrieval can be performed by boiling samples in the target retrieval solution. Heat 700 mL 1× target retrieval solution in a 1 L beaker on a hot plate until a mild boil is reached. Slowly submerge a slide rack with slides into the solution and boil for 10–15 min. Remove slides, rinse them briefly in distilled water and then incubate them in 100% ethanol for 3 min. Proceed to step 8. Do note that this alternative uses more reagents and that bubbles formed during the boiling increases the risk of meninges detaching from slides.

***Note:*** For RNA FISH alone, 10 min of target retrieval is sufficient. However, increasing the duration of this step may improve antibody staining. In our experience, a target retrieval of 15 min yielded improved antibody staining, with no apparent loss of RNA FISH staining, although this may be antibody dependent.

***Note:*** If aiming to perform immunohistochemistry following the RNA FISH protocol, do not let slides dry out after incubation in 100% ethanol. Instead, following ethanol incubation, rinse slides in distilled water. Carefully wipe around the meninges with a piece of paper tissue, making sure not to touch the tissue, and draw the hydrophobic barrier. Then add a small amount of distilled water, without contacting the barrier, to the meninges to avoid them drying out while barrier dries, about 10 min. Continue to step 9.

8. Remove slides from ethanol and dry at RT for 5 min. Using a hydrophobic barrier pen, draw a border around the tissue on the slide. Dry completely at RT, at least 10 min. A continuous barrier is required for steps 9–13 of the RNA FISH protocol and steps 14–18 of the immunohistochemistry protocol.

**Pause point:** It is possible to leave the slides at RT O/N and continue the assay the next day.

***Note:*** Before starting steps 9–12, make sure the hybridization oven is set to 40°C and contains the humidity control tray with a wet filter paper, allow to equilibrate for 30 min. Oven should be set to 40°C for all incubation steps.

9. Transfer slides to the HybEz II hybridization system slide holder. Add 4–5 drops of Protease Plus to each slide and incubate for 15–30 min at 40°C in the hybridization oven. Rinse slides in distilled water. Repeat rinse with fresh distilled water.

***Note:*** During protease incubation, remove desired probes from the fridge, warm to 40°C (about 10 min) in an incubator and then cool to RT.

***Note:*** For RNA FISH alone, 15 min of protease digestion is sufficient. If also performing immunohistochemistry using antibodies, protease digestion time will need to be optimized. In our hands, we achieved the best results using a protease digestion of 15 (anti-CD31 antibody) to 30 min (anti-IBA1 antibody).

10. Remove excess liquid from slides by holding the slide holder vertically and carefully dabbing the lower portion of each hydrophobic area with a piece of tissue.
  a. Add 100–150 μL (4–6 drops) probe mixture to each sample so that the tissue is completely covered.
  b. Incubate in hybridization oven for 2 h.
  c. Wash slides, still locked in slide holder, in 200 mL 1× wash buffer for 2 min with gentle agitation. Repeat wash. Unless otherwise noted, all subsequent washes are performed similarly.

***Note:*** Probes must be mixed to the correct concentration before use. Channel 1 (C1) probes come at a ready-to-use concentration while C2 and C3 probes must be diluted 1:50 in the C1 probe or in probe diluent if no C1 probe is used.

***Note:*** During probe hybridization, remove AMPs and HRPs from the fridge and equilibrate to RT.

11. Hybridize AMPs:
  a. Remove excess liquid from slides and add 4–6 drops AMP1, incubate in the hybridization oven for 30 min.
  b. Wash slides with 1× wash buffer.
  c. Remove excess liquid and add 4–6 drops AMP2. Incubate in hybridization oven for 30 min.
  d. Wash slides with 1× wash buffer.
  e. Remove excess liquid. Add 4–6 drops AMP3 and incubate in hybridization oven for 15 min.
  f. Wash slides with 1× wash buffer.

***Note:*** During AMP3 hybridization, remove desired Opal dye from fridge and equilibrate to RT, in the dark.

12. Develop HRP signal:
  a. Remove excess liquid and add 4–6 drops of the correct HRP (e.g., HRP-C1 if probe is in C1) to the slides.
  b. Incubate in the hybridization oven for 15 min.
  c. Wash slides with 1× wash buffer.
  d. Remove excess liquid, add 100–150 μL of the appropriate Opal dye (as substrate for HRP).
  e. Incubate in the hybridization oven for 30 min.
  f. Wash slides with 1× wash buffer.
  g. Remove excess liquid, add 4–6 drops HRP blocker to each slide.
  h. Incubate in the hybridization oven for 15 min.
  i. Wash slides with 1× wash buffer.
  j. If using more than one probe, repeat step 12 with other HRPs and opal dyes.

***Note:*** Any Opal dye color can be assigned to any probe channel. We have used the Opal 520, Opal 570 and Opal 690 dyes. After being reconstituted following manufacturers’ directions, dyes are used at a dilution of 1:1500, diluted in TSA buffer.

***Note:*** Once opal dyes have been added to the sample, make sure to protect the sample from light as much as possible.

***Note:*** If meninges detach from the microscope slide during the staining, refer to troubleshooting problem 3.

***Optional:*** If desired, perform immunohistochemistry at this point, starting at step 14 below.

13. Counterstain and mount slides:
  a. Remove excess liquid and add a few drops of DAPI to the slide. Incubate for 30 s at RT.
  b. Tap off excess DAPI and mount slides using a coverslip and mounting medium, e.g., ProLong Glass Antifade Mountant.
  c. Let slides cure, protected from light, O/N at RT before viewing.

### Perform optional immunohistochemistry

**Timing: 4 h**

Immunohistochemistry allows for the visualization of proteins in the meningeal whole mounts.

This protocol follows the manufacturer's protocol, with a few modifications, and can be found using the following link (document #323100-TN). https://acdbio.com/sites/default/files/323100-TN%20Multiplex%20Fluorescent%20V2%20with%20IF_incOpal780.pdf

14. Block tissue:
  a. Following the last HRP-blocker step and washes (step 12 above), wash slides two times with TBST wash buffer for two min each.
  b. Add 100–150 μL 10% normal serum diluted in TBSB.
  c. Incubate at RT for 30 min, protected from light, in a humidity chamber.

***Note:*** For optimal blocking, use serum from the same species where the secondary antibody was raised.

15. Add primary antibody:
  a. Tap slides to remove excess blocking solution and add 100–150 μL primary antibody diluted in TBSB within the hydrophobic barrier.
  b. Incubate for 2 h at RT, protected from light, in a humidified chamber.
  c. Wash slides in TBST with gentle agitation three times for 5 min each.

***Note:*** Primary antibody incubation time and dilution should be adjusted to optimize staining and minimize background.

16. Add secondary antibody:
  a. Add 100–150 μL HRP-conjugated secondary antibody diluted in TBSB.
  b. Incubate for 30 min at RT.
  c. Wash slides in TBST with gentle agitation three times for 5 min each.
17. Add Opal reagent:
  a. Add 100–150 μL of the appropriate Opal dye diluted in TSA buffer to each sample.
  b. Incubate for 10 min at RT.
  c. Wash slides in TBST with gentle agitation three times for 2 min each.
18. Counterstain and mount slides as described in step 13 above.

### Image slides

**Timing: 1–4 h**

Image slides in a fluorescence microscope to visualize mRNA and protein expression.

19. Image slides using a fluorescence microscope equipped with an appropriate light source and filter sets. If experiencing problems with RNA FISH or immunohistochemistry signals, refer to troubleshooting problem 4 and problem 5. We commonly use the following setup on a Nikon *Ti2* widefield fluorescence microscope:
  a. Objectives: Plan APO 20×/0.8 NA (air) or Plan Fluor 40×/1.3 NA (oil).
  b. Filter sets:

**Table:** 

Dye	Excitation range (nm)	Emission range (nm)	Common filter set name
DAPI	338–372	414–480	DAPI
Opal 520	443–489	497–551	GFP
Opal 570	540–568	579–640	TRITC
Opal 690	590–645	659–736	Cy5

20. Analyze acquired images using an image analysis program, e.g., Fiji (ImageJ) (Schneider et al., 2012).

## Expected outcomes

**Figure 3:**
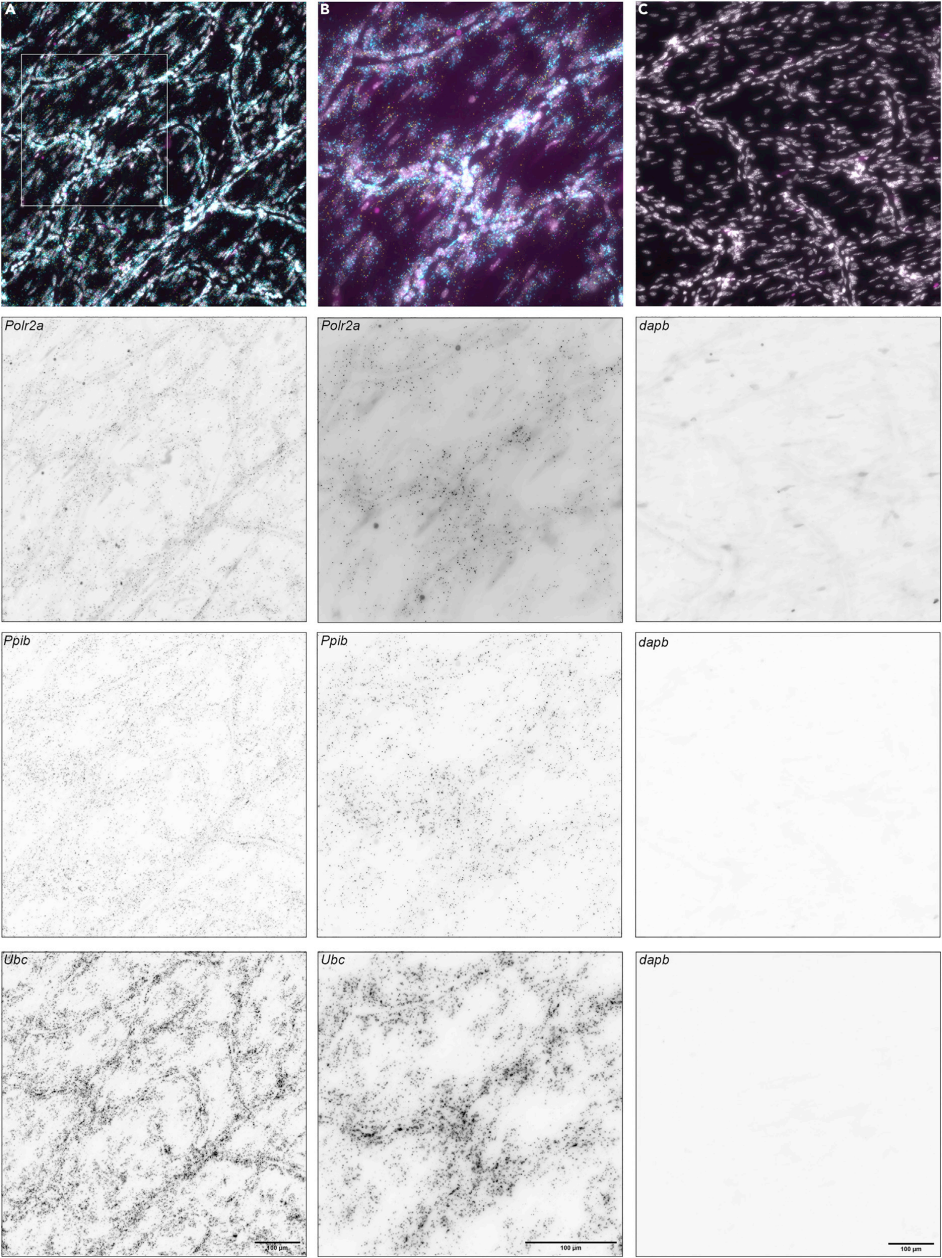
mRNA control probes in dried meningeal whole mounts Positive (A and B) and negative (C) control RNA FISH probes in dried meningeal whole mounts. Dried meningeal whole mounts were stained with probes targeting *Polr2a* (low expression), *Ppib* (medium expression) and *Ubc* (high expression) mRNA. Negative control probe targets *dapB*, an mRNA from *Bacillus subtilis* and thus absent from mouse tissues. Images were captured with a 20× (A and C) or a 40× (B) objective on a Nikon Ti2 widefield fluorescence microscope. Insert in (A) indicates area imaged in (B). In the top panels (merge): *Polr2a* = magenta, *Ppib* = yellow, *Ubc* = cyan, DAPI = gray. Scale bars, 100 μm.

**Figure 4:**
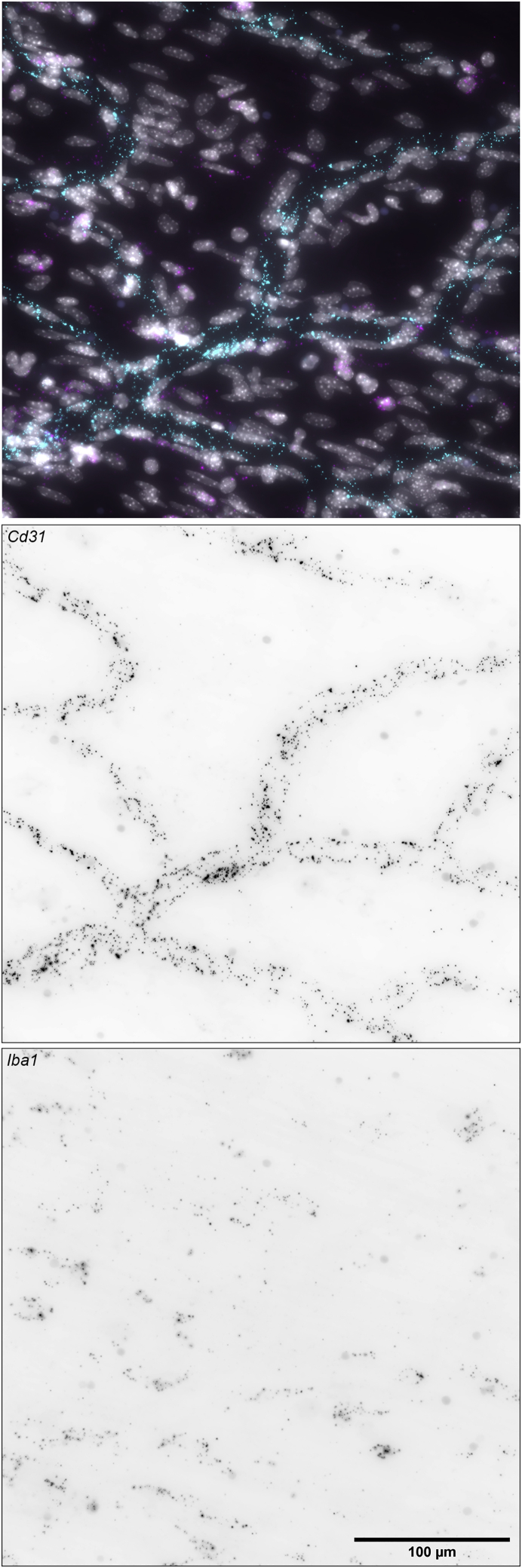
*Cd31* (*Pecam1*) and *Iba1* (*Aif1*) mRNA signal from RNA FISH in dried meningeal whole mounts Images were captured with a 40× objective on a Nikon Ti2 widefield fluorescence microscope. In the top panel (merge): *Cd31* = cyan, *Iba1* = magenta, DAPI = gray. Scale bar, 100 μm.

**Figure 5:**
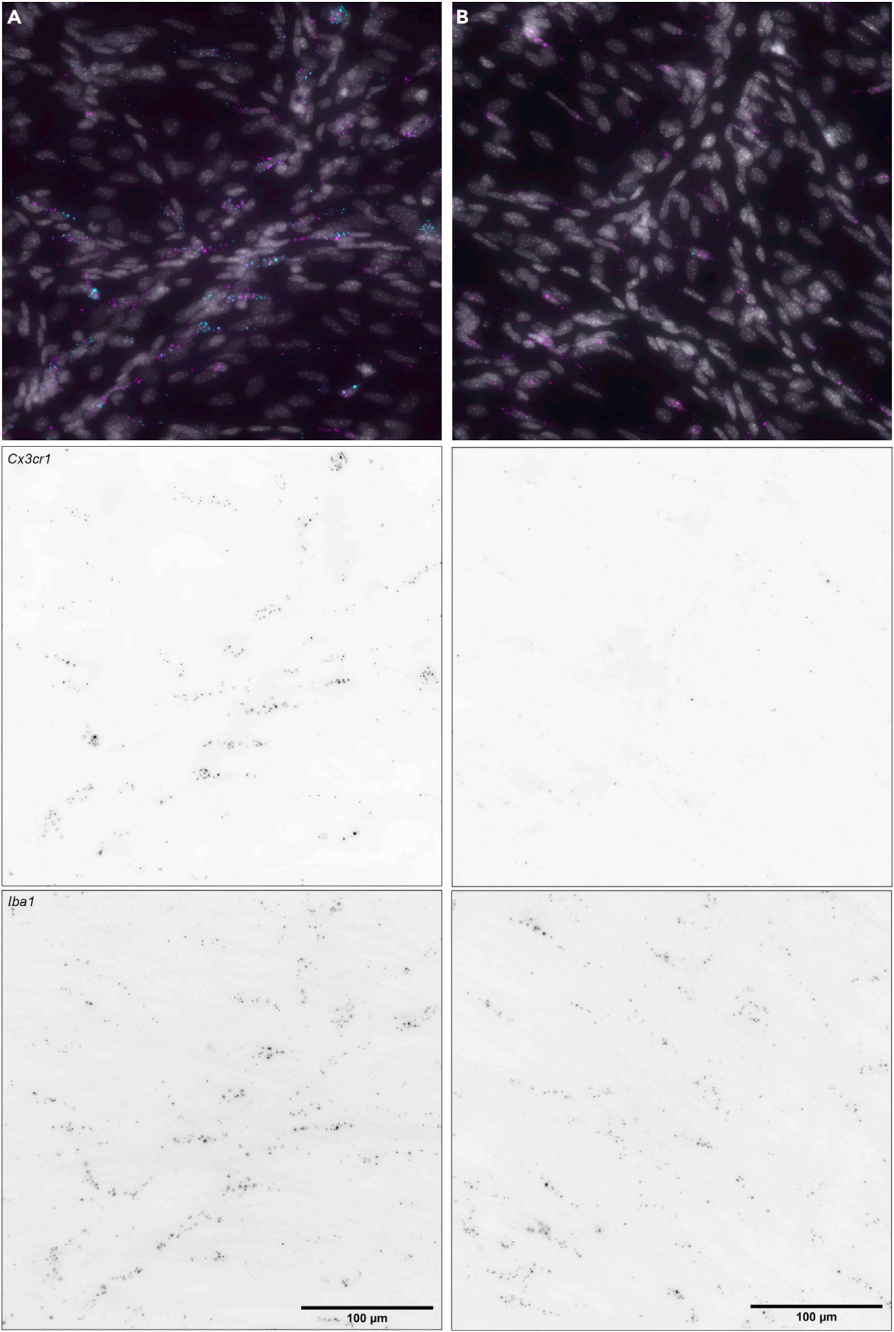
Loss of mRNA signal in KO mice *Cx3cr1* and *Iba1* (*Aif1*) mRNA signal in *Cx3cr1* heterozygous (A) and knock out (B) mice. Notice the greatly reduced *Cx3cr1* signal in the KO mice. Images were captured with a 40× objective on a Nikon Ti2 widefield fluorescence microscope. In the top panel (merge): *Cx3cr1* = cyan, *Iba1* = magenta, DAPI = gray. Scale bars, 100 μm.

**Figure 6:**
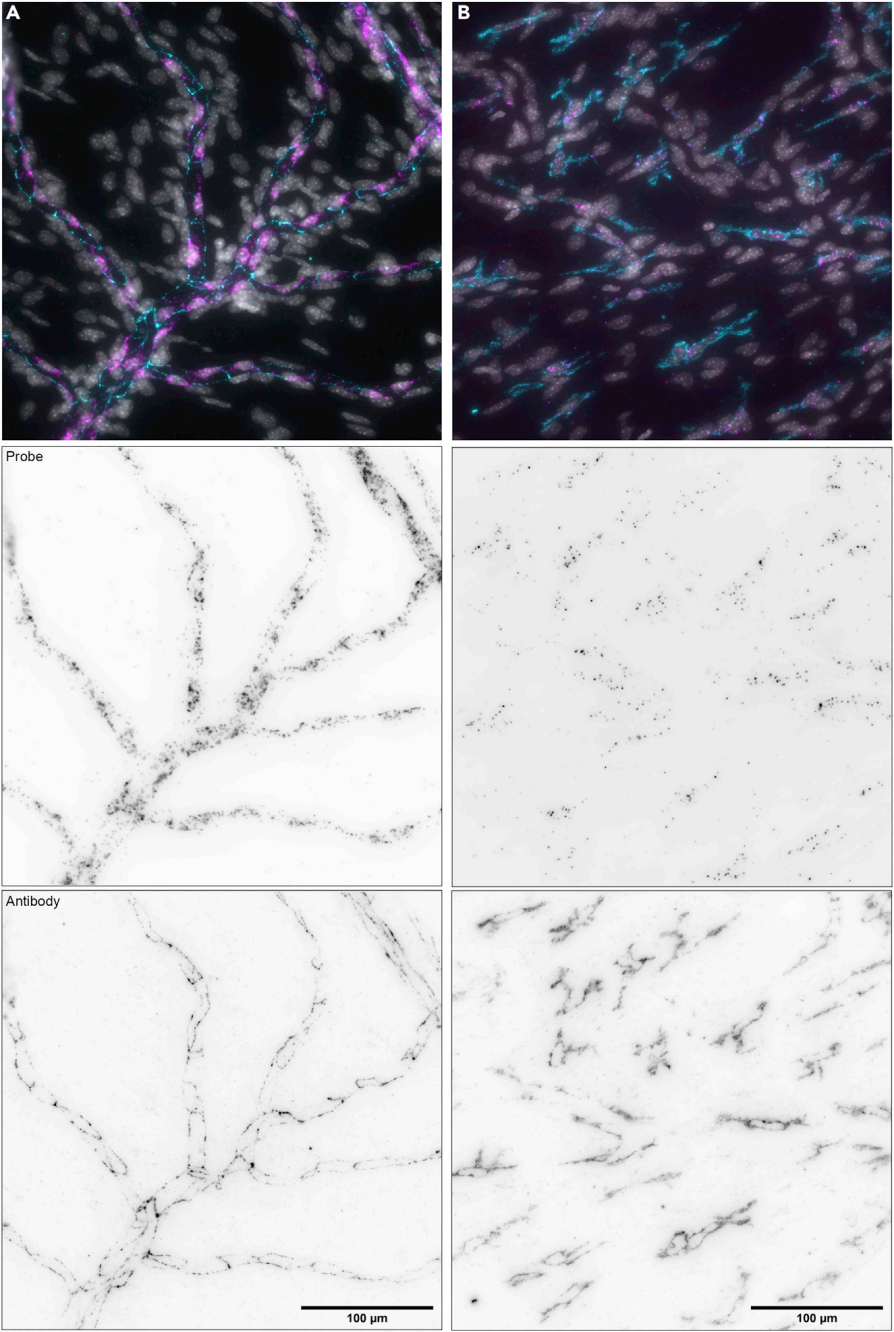
mRNA and protein staining in dried meningeal whole mounts (A) *Cd31* and anti-PECAM-1/CD31 and (B) *Iba1* and anti-AIF1/IBA1. Whole mounts were first subjected to RNA FISH followed by immunohistochemistry targeting the corresponding protein. Notice the overlap of the mRNA and protein signals. Images were captured with a 40× objective on a Nikon Ti2 widefield fluorescence microscope. In top panels (merge): mRNA probe (FISH) signal = magenta, protein (immunohistochemistry) signal = cyan, DAPI = gray. Scale bars, 100 μm.

**Figure 7:**
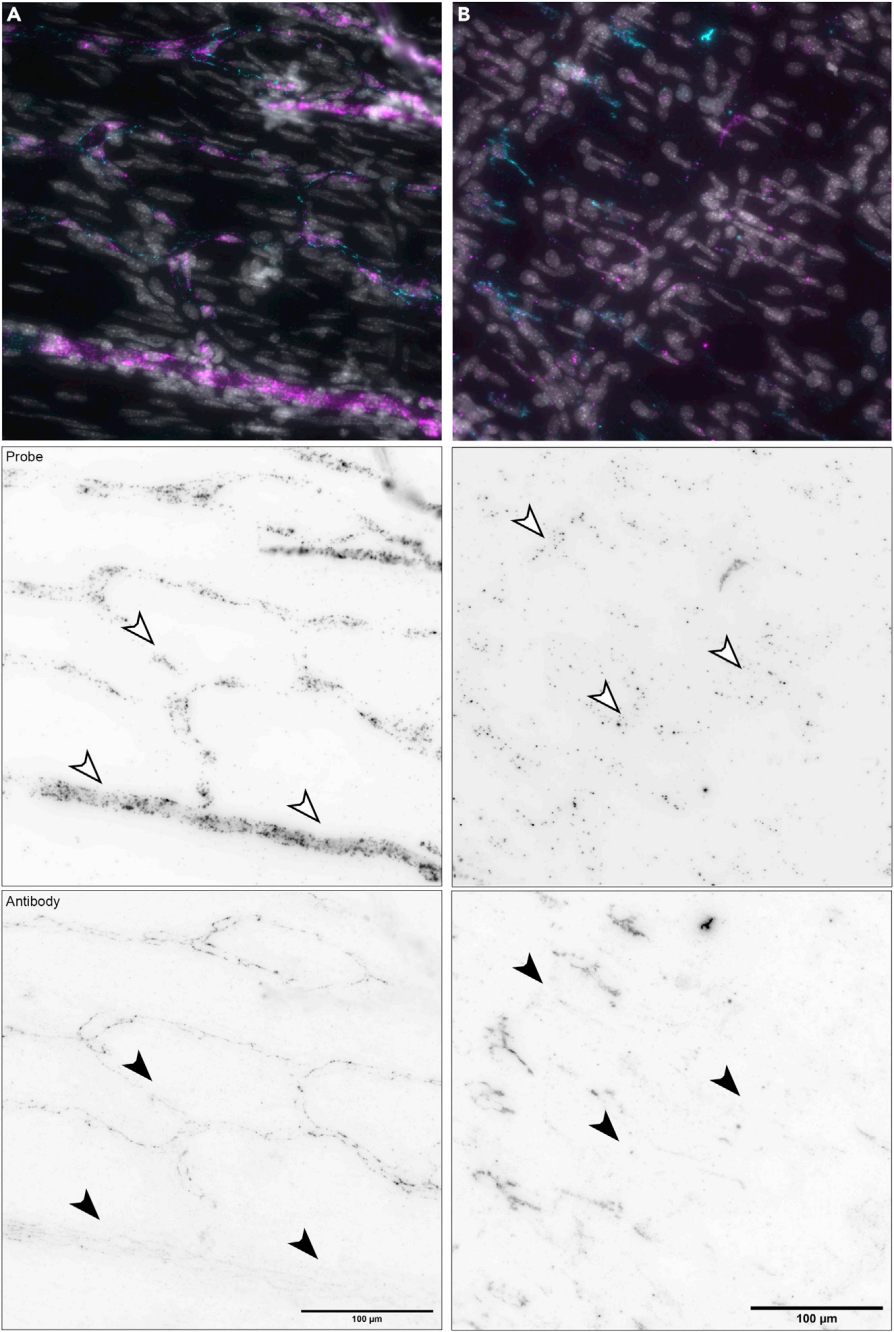
Examples of inconsistent antibody staining with antibodies in the periphery of dried meningeal whole mounts (A) anti-PECAM-1/CD31 and (B) anti-AIF1/IBA1. Samples were first subjected by RNA FISH followed by immunohistochemistry targeting the corresponding protein. Notice absence of antibody staining (filled arrowheads) but not probe staining (open arrowheads) in some areas. Images were captured with a 40× objective on a Nikon *Ti2* widefield fluorescence microscope. In top panels (merge): mRNA probe (FISH) signal = magenta, protein (immunohistochemistry) signal = cyan, DAPI = gray. Scale bars, 100 μm.

Please see figures for expected RNA FISH (Figures 3, 4, and 5) and immunohistochemistry (Figure 6, Figure 7) outcomes. All images were acquired as Z-stacks. The only image processing performed was assembling of images into maximum intensity projections and pseudo coloring of channels in Fiji (Schneider et al., 2012). No alterations of brightness or contrast were performed.

## Quantification and statistical analysis

The punctate mRNA signal produced by the RNAscope assay can be quantified using an image analysis program such as Fiji (ImageJ). We have quantified low and medium expression genes by thresholding the appropriate channel followed by the “analyze particles” function. However, with high expression targets it is often not possible to separate individual objects. Guidelines for how to quantify high expression targets can be found on the ACD website (https://acdbio.com/get-most-out-your-rnascope%C2%AE-multiplex-fluorescent-assays).

**Figure 8:**
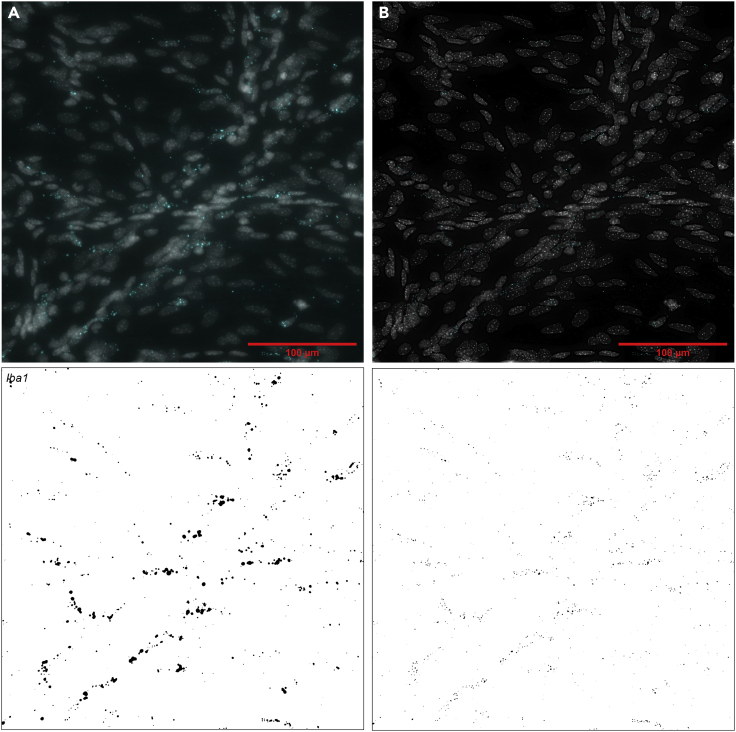
Thresholding of mRNA signal for quantification Examples of thresholding for *Iba1* (*Aif1*) mRNA signals in original (A) and deconvolved (B) images. The original image is the same as in panel A of Figure 3, Figure 4, Figure 5. Image was deconvolved using the SVI Huygens Essential software. Only the DAPI and *Iba1* stain is shown for clarity. Lower panels show thresholding of *Iba1* signal (black). Notice the better separation of objects in the deconvolved image. In top panel (merge): *Iba1* = cyan, DAPI = gray. Scale bars, 100 μm.

It is also worth noting that deconvolution of images captured on a widefield microscope, using e.g., the SVI Huygens software (https://svi.nl/HomePage), can improve quantification by enabling enhanced separation of objects during thresholding (Figure 8). As always when using deconvolution, care should be taken not to lose data.

## Limitations

The RNA FISH part of this protocol is robust and consistently yields good results in our laboratory, showing little to no variation in binding between probes, whereas antibody staining is less predictable and often requires optimization of multiple steps (e.g., target retrieval, protease digestion, antibody dilution, incubation time). Since the protocol requires a protease digestion step, antibodies whose epitopes are sensitive to proteases may not work (Baleriola et al., 2015; Duncan et al., 2015; Kersigo et al., 2018). Furthermore, antibody staining tends to be inconsistent across the dried meninges (Figure 7).

Fluorescent proteins, such as GFP, typically lose their fluorescence during processing for RNA FISH. However, major adjustments to the protocol such as fixation with 4% PFA, omitting the heat-induced target retrieval or lowering protease incubation time may allow the fluorescence of endogenous proteins to be retained (Gross-Thebing et al., 2014; Kersigo et al., 2018) Alternatively, antibodies or mRNA probes targeting fluorescent proteins or their transcription can be used to look for their expression.

## Troubleshooting

### Problem 1

Residual red blood cells present in tissue (step 1).

### Potential solution

Blood and red blood cells can cause significant background fluorescence. Thus, performing an effective perfusion of mice prior to tissue collection is vital to good assay results. While perfusing, look for blanching of the liver and muscle twitching, which indicates draining of the blood. Apply a slow, steady pressure on the syringe. Too high pressure can damage vessels and lead to artifacts.

Conditions causing clotting or activation of the blood vessel endothelium, such as bacteremia, will impair the perfusion as blood cells will remain attached in blood vessels. This is mostly unavoidable, depending on the nature of the study.

### Problem 2

Meninges detach from microscope slides during target retrieval (step 7).

### Potential solution

Meninges must be properly dried to stay attached during the target retrieval. For the microscope slides utilized in this protocol, 3 days at RT (temperature and humidity range over 24 h of 20°C–21°C and 25%–30%, respectively) or 24 h at 42°C are required for proper attachment (Figure 2). Furthermore, it is crucial that meninges are completely flat on the microscope slide as wrinkles or bubbles may cause them to detach during target retrieval and washes (Grabinski et al., 2015). Aim to dry meninges with the ventral side facing upwards, as this will minimize any space forming between the tissue and the microscope slide.

Even if dried as outlined in this protocol, prolonged target retrieval will cause meninges to detach. In our hands, target retrieval longer than 15–20 min causes partial detachment and 30 min results in almost complete detachment from slides.

### Problem 3

Meninges detach from microscope slides during staining (steps 10–12).

### Potential solution

Similar to how meninges may detach during target retrieval, they may also detach or lose integrity during the staining and wash steps of the protocol. As in problem 2, make sure meninges are dried appropriately on the microscope slide, taking care to avoid wrinkles and bubbles forming in the tissue. Prolonged incubation time with protease may cause degradation of the tissue and detachment from the microscope slide.

Take care to perform wash steps with very gentle and slow agitation of the slides. Intense rocking of the slides will eventually cause the sides of the tissue to detach from the microscope slide.

### Problem 4

Low signal for mRNA probe. Hard to separate signal from autofluorescence (step 19).

### Potential solution

In general, mRNA probes produce a strong signal, visible as distinct puncta in the meningeal tissue. Signal strength depends on the number of ZZ target probes binding to the target mRNA and if a probe contains a low number of ZZ pairs, signal will be weaker. To increase signal strength, make sure to use optimal assay conditions.

RNA degradation within the sample or the provided probes has not been a problem in our hands. Probes have a shelf life of 2 years when stored as recommended.

Autofluorescence is relatively high in meningeal tissue, especially in the green range of the visible spectrum (see second row panels in Figure 3). Since any color can be assigned to any probe channel, try to use an Opal dye in the red or far-red channels, where autofluorescence is less of a problem. Alternatively, try using a bright probe or highly expressed mRNA target in the green channel. Lastly, red blood cells are highly autofluorescent, thus it is vital to exsanguinate mice completely (by flushing with a minimum of 10 mL saline) before fixation.

### Problem 5

Unspecific, weak or non-existent antibody staining (step 19).

### Potential solution

The processes of fixation, drying, target retrieval and protease digestion can alter the epitopes recognized by antibodies (Ge et al., 2006; Gross-Thebing et al., 2014; Ramos-Vara and Miller, 2014; Young et al., 2020). Thus, the protocol presented here will need to be optimized for use with antibodies other than the ones described herein. Even with optimization, some areas of the meninges may display inconsistent antibody staining, usually on the edges of the sample (Figure 7).

In our experience, key parameters to adjust are the target retrieval and protease digestion times. Try varying the target retrieval time from 10 to 20 min. A longer target retrieval may not be possible due to detachment of meninges from slides. A shorter target retrieval may result in reduced mRNA signal. Digestion times can be varied from 15 min up to 30 min without loss of mRNA signal and may increase antibody signal. However, a protease digestion time of 45 min or more will result in loss of mRNA signal.

If unspecific staining is encountered, try diluting the primary antibody more. This may reduce off target staining.

Following target retrieval and incubation in 100% ethanol, do not let slides dry out if performing immunohistochemistry. Keeping slides wet will make staining more consistent for some antibodies.

While it is possible to use secondary antibodies conjugated to fluorophores, we recommend using an HRP-conjugated secondary antibody in combination with one of the Opal dyes, which are based on tyramide signal amplification and yields strong fluorescence signals (Kerstens et al., 1995; van Gijlswijk et al., 1997). This has consistently worked well in our hands.

## Resource availability

### Lead contact

Further information and requests for resources and reagents should be directed to and will be fulfilled by the lead contact, Olivia Steele-Mortimer (omortimer@niaid.nih.gov).

### Materials availability

This study did not generate new unique reagents.

## Data Availability

All data reported in this paper will be shared by the lead contact upon request. This paper does not report original code.

## Supplemental information

Document S1. ▪ ▪ ▪
